# A KEY DENDRITE DEVELOPMENT REGULATOR PLEXIN-A4 IS ASSOCIATED WITH DENDRITE MAINTENANCE IN AGED MICE

**DOI:** 10.1093/geroni/igad104.3139

**Published:** 2023-12-21

**Authors:** Jiyeon Baek, Haniya Naveed, Laura Muyingo, Angélica Jimenez Romero, Carol Eisenberg, Jack DeLucia, Tracy Tran

**Affiliations:** Rutgers, The State University of New Jersey, Newark, New Jersey, United States; Rutgers, The State University of New Jersey, Newark, New Jersey, United States; Rutgers, The State University of New Jersey, Newark, New Jersey, United States; Heritage University, Toppenish, Washington, United States; Rutgers, The State University of New Jersey, Newark, New Jersey, United States; Rutgers, The State University of New Jersey, Newark, New Jersey, United States; Rutgers, The State University of New Jersey, Newark, New Jersey, United States

## Abstract

There is significant loss of dendritic complexity with age, which may account for synaptic loss and cognitive decline in mammals. However, molecular mechanisms underlying these changes are unclear. Key molecular players in dendritic morphogenesis include the guidance cue semaphorin 3A (Sema3A). In developing cortical neurons, Sema3A signals through the Plexin-A4 receptor to induce basal dendritic arborization, while another closely related member, Sema3F, induces apical dendritic spine pruning. Interestingly, both ligands are expressed in adulthood, but their roles in age-associated dendritic remodeling are unknown. To address this, we established a model of aging mouse primary cortical neurons: 5, 15, and 30 days-in-vitro (DIV) representing young, mature, and aged timepoints. Treating with either ligand increases branching at 5-15DIV, but not 30DIV, suggesting age-associated changes in cellular responses to Sema3A/3F. Next, we examined mouse cortices in vivo and found both Plexin-A4 expression and dendritic elaboration decrease with age, suggesting a need for Plexin-A4 to maintain dendritic complexity at older ages. In 6-month-olds, mutant Plexin-A4 mice have less branching than wildtypes, but we found no further decline in 18-month-olds, likely because there can be no age-associated downregulation of Plexin-A4 signaling if the receptor is not present. Finally, we found increased levels of active-RhoA GTPase (a downstream cytoskeleton regulator) in Plexin-A4-/- 6-month-old cortices versus controls. Additionally, our preliminary data trends towards increased active-RhoA in 18-month-olds versus 6-month-olds. Collectively, our study provides novel insights into the diverse cellular functions of semaphorin-plexin, a signaling pathway important for nervous system development, in age-associated dendritic loss and cognitive decline.

